# The pattern‐recognition molecule mindin binds integrin Mac‐1 to promote macrophage phagocytosis via Syk activation and NF‐κB p65 translocation

**DOI:** 10.1111/jcmm.14236

**Published:** 2019-03-14

**Authors:** Yuan‐sheng Liu, Li‐fen Wang, Xiao‐Shen Cheng, Ya‐Ni Huo, Xiao‐Mei Ouyang, Lai‐Ying Liang, Ying Lin, Jian‐Feng Wu, Jian‐Lin Ren, Bayasi Guleng

**Affiliations:** ^1^ Department of Gastroenterology Zhongshan Hospital affiliated to Xiamen University Xiamen China; ^2^ The Second Affiliated Hospital of Guangzhou Medical University Guangzhou China; ^3^ State Key Laboratory of Cellular Stress Biology, School of life sciences Xiamen University Xiamen China; ^4^ Faculty of Clinical Medicine & Institute of Microbial Ecology Medical College of Xiamen University Xiamen China

**Keywords:** Mac‐1, mindin, phagocytosis

## Abstract

Mindin has a broad spectrum of roles in the innate immune system, including in macrophage migration, antigen phagocytosis and cytokine production. Mindin functions as a pattern‐recognition molecule for microbial pathogens. However, the underlying mechanisms of mindin‐mediated phagocytosis and its exact membrane receptors are not well established. Herein, we generated mindin‐deficient mice using the CRISPR‐Cas9 system and show that peritoneal macrophages from mindin‐deficient mice were severely defective in their ability to phagocytize *E  coli*. Phagocytosis was enhanced when *E  coli* or fluorescent particles were pre‐incubated with mindin, indicating that mindin binds directly to bacteria or non‐pathogen particles and promotes phagocytosis. We defined that ^131^I‐labelled mindin binds with integrin Mac‐1 (CD11b/CD18), the F‐spondin (FS)‐fragment of mindin binds with the α_M_‐I domain of Mac‐1 and that mindin serves as a novel ligand of Mac‐1. Blockade of the α_M_‐I domain of Mac‐1 using either a neutralizing antibody or si‐Mac‐1 efficiently blocked mindin‐induced phagocytosis. Furthermore, mindin activated the Syk and MAPK signalling pathways and promoted NF‐κB entry into the nucleus. Our data indicate that mindin binds with the integrin Mac‐1 to promote macrophage phagocytosis through Syk activation and NF‐κB p65 translocation, suggesting that the mindin/Mac‐1 axis plays a critical role during innate immune responses.

## 1 INTRODUCTION

The mononuclear phagocyte system is composed of monocytes circulating in the blood and macrophages that infiltrate tissues and organs. Phagocytes are one of the main types of antigen presenting cells in the body and have been demonstrated to be important components of innate immunity.^1^, ^2^ In response to infection, monocytes firmly adhere to blood vessels, transmigrate through the endothelial layer and differentiate into macrophages that phagocytose microbial pathogens.^3^, ^4^ Two methods are utilized by macrophages to eliminate foreign microbial pathogens. In one method, macrophages directly interact with bacteria to promote phagocytosis, and in the other method, cytokines and complement factors function as opsonins to enhance macrophage phagocytosis.^4^ An opsonin needs to recognize and react with foreign antigens and then interact with macrophage receptors to initiate an immune response. Examples of receptors include complement receptors that react with C3b, C4b and iC3b, the Fc receptor (FcR) that reacts with immunoglobulins (Ig) and the C1q receptor that reacts with lectin.^5^, ^6^

Integrin Mac‐1 (CR3, CD11b/CD18, α_M_β_2_) is a β_2_ integrin receptor with broad ligand binding specificity that is expressed on neutrophils and monocytes/macrophages.^7^ Similar to other β_2_ integrins, Mac‐1 binds ligands through the α‐chain I domain (α_M_I).^8^, ^9^ α_M_β_2_ is reported to be the most promiscuous integrin, with more than 40 ligands, including intercellular adhesion molecule‐1 (ICAM‐1) on inflamed endothelial cells, the complement protein C3bi, fibrinogen, fibrin, collagen and coagulation factor X.^10^, ^11^ Integrin α_M_β_2_ supports various adhesive functions, including leukocyte recruitment, pathogen clearance, antigen presentation, and thrombosis.^7^, ^8^

Mindin (also known as spondin 2) is a highly conserved extracellular matrix protein that contains F‐spondin domains 1 and 2 (FS1 and FS2) at the N‐terminus and thrombospondin type 1 repeats (TSR) at the C‐terminus.^12^, ^13^ We recently reported that mindin attenuates colon cancer progression by blocking angiogenesis via Egr‐1‐mediated regulation.^14^ In addition, as a pattern‐recognition molecule, mindin directly binds to bacteria and promotes clearing influenza viruses and bacteria.^15^, ^16^ The ability to eliminate invading microbial pathogens and respond to a broad spectrum of microbial stimuli is impaired in mindin‐deficient mice.^16^ Previous studies have concluded that mindin acts as an opsonin for macrophage phagocytosis.^16^ However, the mechanisms of mindin‐mediated phagocytosis are not well understood. It has demonstrated that the αMβ2 and α4β1 integrins are mindin ligands on neutrophils and the FS domain of mindin binds to the αMβ2 integrin.^17^, ^18^ They also reported that the mindin‐integrin interaction is critical for inflammatory cell recruitment in vivo.^18^ However, with regard to another important phagocytotic function of macrophages, does not have direct evidence of participation in the mindin‐integrin interaction.

Herein, we generated mindin‐deficient mice using the CRISPR‐Cas9 system and show that peritoneal macrophages from mindin‐deficient mice are severely defective in their ability to phagocytize *E  coli*. We also confirmed that the FS fragment of mindin binds with the αM‐I domain of Mac‐1. Our data indicate that the mindin‐integrin interaction was involved in phagocytosis of macrophages. Blockade of the αM‐I domain of Mac‐1 reduces the mindin‐induced phagocytosis. Mindin also activates the Syk family and translocates NF‐κB p65 into the nucleus. Our results suggest that mindin is a pattern‐recognition molecule that binds with integrin Mac‐1 to promote phagocytosis of macrophages through Syk activation and NF‐κB p65 translocation.

## 2 MATERIALS AND METHODS

### 2.1 Ethics statement

All procedures involving experimental animals were performed in accordance with protocols that were approved by the Committee for Animal Research of Xiamen University and complied with the Guide for the Care and Use of Laboratory Animals (NIH publication No. 86‐23, revised in 1985).

### 2.2 Generation of mindin knockout mice using a CRISPR‐Cas9 system

This experiment was performed in collaboration with the State Key Laboratory of Cellular Stress Biology at Xiamen University. Generation of mindin knockout mice was performed according to Zhang et al.^19^ Injection of fertilized eggs was performed according to Mikhaleva et al.^20^ Briefly, we designed the first exon gRNA sequence (5'– GCGGAAGAATGTATGTAAG–3') for mindin based on the online software developed by Professor Zhang Feng at Massachusetts Institute of Technology (MIT). The corresponding gRNA plasmids, which were constructed using T4 DNA ligase (Takara, Dalian, China) and pSpCas9(BB)‐2A‐GFPpUC19 (a gift from the State Key Laboratory of Cellular Stress Biology at Xiamen University), were transferred into murine cells (L929). The most efficient gRNA sequences were screened (the sequence is marked with a black box in Figure S1) and transferred to a plasmid vector expressing a T7 promoter. The pSpCas9(BB)‐2A‐GFPpUC19 and the gRNA plasmid with the T7 promoter were digested and recovered by phenol/chloroform extraction and were resuspended in nuclease‐free water. The transcribed RNAs, which were transcribed using a Sp6 mMESSAGE mMACHINE Kit (Ambion, Carlsbad, CA), were diluted at a concentration of 20 ng/µL and injected 2pl into fertilized eggs of C57BL/6 J mice using an injection needle. After injection, the fertilized eggs were returned to surrogate mothers. Mice were housed in a specific‐pathogen‐free (SPF) facility. Newborn mouse tail DNA samples were amplified with PrimeStar (Takara, Dalian, China), and the PCR products were sequenced for screening of heterozygous or knockout descendants using the following primers: (forward) 5'‐ ATACCCTCTCCCAGGCTAGC‐3' and (reverse) 5'‐ CTTTGCTGAGCGTGGTGAGG −3'.

### 2.3 Cell lines and culture conditions

Human embryonic kidney (HEK293T) cells and murine RAW264.7 macrophages were purchased from ATCC (Manassas, VA) and maintained in DMEM (Life Technologies, Beijing, China) supplemented with 10% foetal bovine serum and 1% penicillin G/streptomycin (Life Technologies, Grand Island, NY). Peritoneal macrophages were isolated from wild‐type and mindin‐deficient mice. Briefly, three days after intraperitoneal injection with 4% thioglycollate (Sigma‐Aldrich, St. Louis, MI) into wild‐type or mindin‐deficient mice, macrophages were separated from the intraperitoneal cavity by douching with 5 mL of DMEM containing 10% foetal bovine serum. The cells were maintained in DMEM supplemented with 10% foetal bovine serum and antibiotics. Stable Mac‐1‐overexpressing cells were established by our lab. Briefly, HEK293T cells were transfected with mouse CD11b and CD18 vectors using Lipofectamine 2000 (Invitrogen, Carlsbad, CA) and were selected with DMEM containing 1.5 µg/mL puromycin (Invitrogen, Carlsbad, CA) for 2 weeks. The puromycin‐resistant cell clones were collected and analysed via immunoblotting for stable expression of Mac‐1.

### 2.4 Antibodies and other reagents

Mouse recombinant mindin protein was purchased from R&D Systems (Minneapolis, MN). The following antibodies were used: rabbit monoclonal anti‐GAPDH and CD11b antibodies [cat no: 20991‐1‐AP] (Proteintech, Rosemont, Pennsylvania); mouse anti‐Myc‐tagged antibody (Proteintech); rabbit monoclonal anti‐phospho‐MEK1/2, anti‐MEK1/2, anti‐phospho‐Zap70 and anti‐NF‐κB p65 antibodies (Cell Signalling, Beverly, MA); rabbit monoclonal anti‐phospho‐SykY323, anti‐phospho‐SykY525/526, anti‐Syk, anti‐CD11b [clone no: M1/70] and anti‐CD11b [cat no:ab128797] antibodies (Abcam, Cambridge, MA) and mouse monoclonal anti‐CD18 [clone no: M18/2] antibody (Abcam); mouse monoclonal anti‐CD18 antibody [cat no: CTB104], rabbit and mouse IgG antibodies and anti‐HA antibodies (Santa Cruz Biotechnology, Santa Cruz, CA); fluorescence isothiocyanate (FITC)‐labelled goat anti‐rabbit secondary antibody and tetramethyl rhodamine isothiocyanate (TRITC)‐labelled goat anti‐mouse secondary antibody (Cell Signalling, Beverly, MA); mouse polyclonal anti‐PARP antibody (BioSource, Camarillo, CA); rabbit monoclonal anti‐phospho‐ERK1/2 antibody and ERK1/2 antibody (Promega, Madison, WI);pHrodo E.coli (Life Technologies, Grand Island, NY);Phalloidin (Solarbio ShangHai China).

### 2.5 Labelling of bacteria

*E  coli* bacteria were cultured for 16 hours at 37°C in LB broth with FITC (Sigma, St. Louis, MO) at a concentration of 50 µg/mL *E  coli* were then washed twice in PBS and fixed with 4% formaldehydum polymerisatum according to the standard fixative. The organisms were examined by fluorescence microscopy for uniformity of FITC staining. Confirmation was provided by flow cytometry (FCM). The bacteria were suspended in PBS to a final concentration of 10^9^ bacteria per mL and stored at 4°C in a dark environment.

### 2.6 Phagocytosis assay

For phagocytosis experiments, 1 × 10^5^ peritoneal macrophages or RAW264.7 cells were placed in 6‐well plates. After 8 hours, 10 µL of fluorescent particles (1 × 10^9^/mL) (Promega, Madison, WI) or labelled bacteria (1 × 10^9^/mL) or pHrodo E.coli (1 × 10^9^/mL) were added to the 6‐well plates. After incubation for 2.5 hours at 37°C, non‐phagocytosed particles and bacteria were separated from macrophages by washing with 1 mL of PBS three times and phagocytosed beads were counted using either a Leica DM4000 B microscope (Leica Microsystems, Buffalo Grove, IL) or FCM. Phagocytic index = (% of macrophages containing at least two bacterium) × (mean number of bacteria per positive cell). In the inhibition experiments, RAW264.7 macrophages were pre‐treated with neutralizing antibodies: CD11b ([M1/70], ab128797, 20991‐1‐AP)(1:100), CD18 ([M18/2], CTB104)(1:100) for 30 minutes at 37°C and R406(5 µmol/L) and QNZ(3 µmol/L) for 1 hour at 37°C and the same methods were performed as described above. All experiments included blank controls to establish a negative control group, but some of the results from the negative controls are presented in Supplemental Figures.

### 2.7 Flow cytometry

Flow cytometry was performed with a FACSCaliber and LSRFortessa flow cytometer (BD Bioscience, San Diego, CA) using the 488‐nm line of an argon ion laser. Green fluorescence was collected using a 530 ± 15 nm bandpass filter and linear amplification. Red fluorescence was collected using a 560 ± 15 nm bandpass filter and linear amplification. The data were collected and analysed using FlowJo software (Tree Star, Ashland, OR).

### 2.8 Giemsa stains

Briefly, 1 × 10^5^ peritoneal macrophages were placed in a Millicell ZE slide (Millipore, Hong Kong, China). CRBC, fluorescent particles and *E  coli* were added to the plates and incubated for 2.5 hours at 37°C. After three washes with PBS, the cells were fixed with methanol and stained with Giemsa stain (Sigma‐Aldrich, St. Louis, MI). The nuclei and bacteria were stained purple/blue, the cytoplasm of CRBC was stained light blue, and the fluorescent particles were not stained.

### 2.9 Binding assays

Either 2 μL of rMindin, FBS, or LPS was added to a tube with 10 µL of fluorescent particles (1 × 10^9^/mL). After incubation for 30 minutes at 37°C, the mixture was centrifuged at 5000 × *g* and the supernatant was discarded. The precipitate was washed with PBS two times and added to loading buffer for Western blot analysis.

### 2.10 Preparation of ^131^I‐Mindin

In a 1‐mL vial, 10 μg of recombinant mindin protein was dissolved in 100 μL of PBS (0.5 mol/L phosphate buffer, pH 7.4) followed by addition of Na^131^I (approximately 5 mCi). Then, 50 μL of chloramine‐T (1 mg/mL) that had been freshly prepared in water was added. The reaction mixture was allowed to stand for 3 minutes at room temperature. Then, the reaction was terminated by adding 50 μL of Na_2_S_2_O_5_ (2 mg/mL, freshly prepared in water). After purification using Sephadex G25 resin, the RCP and SA of radioiodinated mindin were tested by TLC (polyamide film/saline) and then diluted in PBS for cell uptake and biodistribution experiments.

### 2.11 Mindin uptake assay

To determine the binding of ^131^I‐mindin in RAW264.7 macrophages and stably expressing Mac‐1 cells, 200 μL of ^131^I‐mindin (approximately 0.15 kBq/100 μL) was added to cells (3 × 10^4^) plated on 48‐well plates. After different incubation periods, the supernatants were removed and cells were washed by PBS for three times. Then collected in 1 mol/L NaOH and radiation levels were examined with a γ‐counter. The cell uptake percentage was calculated. The resulting values are expressed as the means ± SD.

### 2.12 Biodistribution

Six‐week‐old male athymic BALB/c nude mice were housed under SPF conditions. HEK293T control cells and HEK293T cells stably expressing Mac‐1 were injected intradermally into each flank of the nude mice and were allowed to grow for 1 week. The mice (n = 3 per group) were injected with ^131^I‐mindin (approximately 40 kBq/100 μL) via the lateral tail vein. At 4 hours or 24 hours post‐injection, organs and tissues were collected, weighed and counted with the γ‐counter. The percentage of the injected dose per gram of tissue (%ID/g) was calculated. The resulting values are expressed as the means ± SD.

### 2.13 Western blot analysis

Total protein was extracted from cells and tissues using the RIPA buffer (Solarbio, Beijing, China) and the cytoplasmic and nuclear proteins for the nuclear extraction kit (Cayman Chemical, Ann Arbor, MI) according to the manufacturer's protocol. Equal amounts of total protein were separated by 10% SDS‐PAGE and then transferred to PVDF membranes. The membranes were blocked with 5% non‐fat milk at room temperature for 1 hour and then incubated with the appropriate primary antibodies overnight at 4°C. The membranes were subsequently incubated with horseradish peroxidase‐conjugated antibodies and visualized with an enhanced chemiluminescence reagent (Millipore, Wanchai, Hong Kong).

### 2.14 RNA extraction and quantitative PCR

In total 3 × 10^5^ of RAW264.7 cells were grown in 6‐well plates, transfected with control or si‐CD18 or si‐CD11b RNA plasmids cultured for an additional 24. The TRIzol reagent (Invitrogen, Carlsbad, CA) was applied to isolate the total RNA of cells and tissues. We synthesized the complementary DNA using a first‐strand reverse transcription kit (Thermo Fisher, Carlsbad, CA), and performed the qPCR using SYBR Green Dye mix (Takara, Kusatsu, Shiga). The following primers were applied to quantitatively analyse the expression of mRNA: CD18 forward for 5′‐ CAGGAATGCACCAAGTACAAAGT −3′ and reverse for 5′‐ CCTGGTCCAGTGAAGTTCAGC −3′, CD11b for 5′‐ ATGGACGCTGATGGCAATACC‐3′ and 5′‐ TCCCCATTCACGTCTCCCA‐3′ and GAPDH for 5′‐ AGGTCGGTGTGAACGGATTTG‐3′ and 5′‐ TGTAGACCATGTAGTTGAGGTCA‐3′. We performed the PCR using 7500 RT‐PCR System (Applied Biosystems, Life Technologies, Carlsbad, CA, USA).

The siRNAs targeting mouse CD18, CD11b and NC siRNA (si Control) were purchased from RiboBio (Guangzhou, China). All oligonucleotide sequences are listed below.

Si‐CD18‐ctrl:5′‐ ATCATTTGACTGGTACATCAA‐3′

Si‐CD18‐1:5′‐CCCAATGTGGCTGCCATCGTA‐3′

Si‐CD18‐2:5′‐ ATCAAGAATGCCTACTATAAA −3′

Si‐CD18‐3:5′‐ CAGACAGAGGTCGGCAAGCAA −3′

Si‐CD11b‐ctrl:5′‐ AGCATGAGTTATCATAATCAA −3′

Si‐CD11b‐1:5′‐ GCACTGAGATCCTGTTTAA‐3′

Si‐CD11b‐2:5′‐ GGAGAATACTTATGTGAAT‐3′.

Si‐CD11b‐3:5′‐ GCAGCCAGATTGGCTCTTA‐3′.

### 2.15 Co‐immunoprecipitation assay

The Mindin and Mac‐1 overexpression vector was constructed as previously described.^14^ HEK293T cells stably expressing Mac‐1 were transfected with plv‐Myc or mindin‐Myc for 48 hours. Whole‐cell extracts were harvested with RIPA buffer (Solarbio, Beijing, China) and were then incubated with 10 µg of protein G‐agarose (Santa Cruz Biotechnology, Santa Cruz, CA) and 3 g of anti‐Myc or anti‐HA antibody overnight at 4°C. After incubation, the beads were washed three times with 500 µL of RIPA buffer. Antibody‐bound complexes were eluted and denatured by boiling in sample buffer. Proteins were analysed by immunoblotting.

### 2.16 Confocal immunofluorescence microscopy analysis

HEK293T cells stably expressing Mac‐1 were transfected with a mindin‐Myc expression vector. After incubation for 48 hours, the cells were fixed with 4% formaldehyde, permeabilized with PBS containing 0.5% Triton X‐100 and blocked with 5% bovine serum albumin (BSA). Cells were immunostained with antibodies against HA and Myc overnight at 4°C with PBS containing 0.5% Triton X‐100. The antigen‐primary antibody complexes were detected using FITC‐labelled goat anti‐rabbit secondary antibody (1:1000) and TRITC‐labelled goat anti‐mouse secondary antibody (1:1000). Cell imaging was performed using an LSM 510 SYSTEM microscope (Zeiss, Germany), and the data were analysed using MetaMorph/MetaFluor version7.0 software (Orleans, Sunnyvale, CA).

### 2.17 Statistical analysis

The results were analysed using SPSS 13 statistical software (IBM, Chicago, IL). Quantitative variables between two groups were compared using Student's *t* test or among multiple groups using one‐way analysis of variance (ANOVA). The correlations were analysed using Spearman's rank test. The differences were considered significant at *P* < 0.05.

## 3 RESULTS

### 3.1 Phagocytosis of different immunogens in mindin‐deficient mice

**Figure 1:**
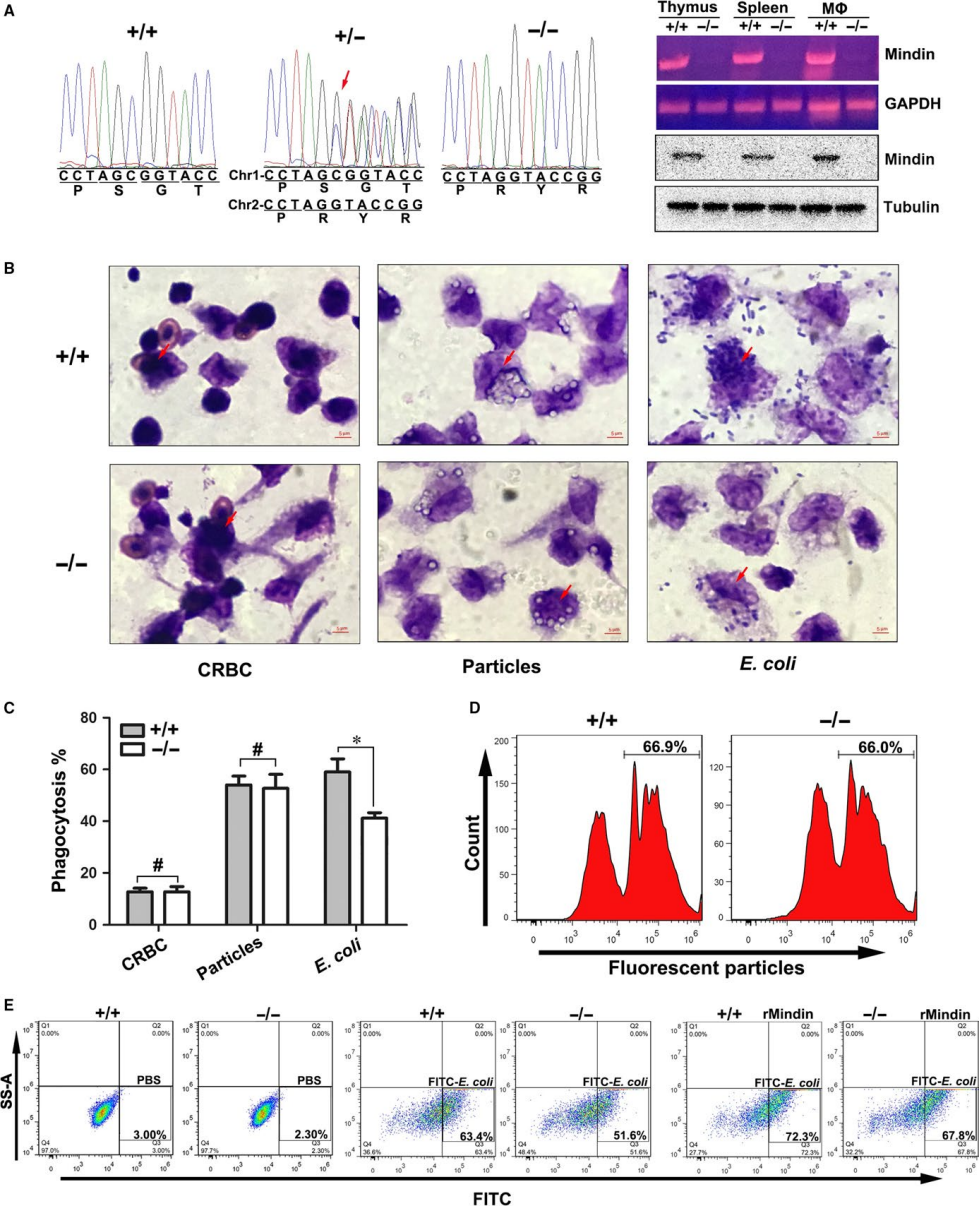
Phagocytosis of different immunogens in mindin‐deficient mice. (A) Sequencing chromatograms show the nucleotide mutation for mindin‐/‐ mice using a CRISPR‐Cas system. The arrow indicates a double peak. RT‐PCR and Western blot analysis of mindin protein levels in the thymus, the spleen and peritoneal macrophages (MΦ) obtained from wild‐type and mindin‐/‐mice. (B) The phagocytosis response to different immunogens was analysed with Giemsa staining, and the arrow indicates the phagocytized cells. The data are presented as the percentage of different immunogens that were phagocytosed compared among all the groups and are shown the means ± SD from three separate experiments. *P < 0.05, #no significant difference (C). Peritoneal macrophages obtained from wild‐type and mindin^‐/‐^ mice were incubated with fluorescent particles (D) or PBS or *E  coli *or mixtures of *E  coli* and rMindin (E) for 2.5 h. Phagocytosis was measured by flow cytometry

The immunogens that cause human inflammation include three major categories: foreign tissue, fine particles and microbial pathogens.^4^, ^21^ To investigate whether mindin has the ability to induce macrophage phagocytosis for all types of immunogens, we designed and generated mindin knockout mice using a CRISPR‐Cas system (Figure 1A) and isolated peritoneal macrophages of wild‐type and mindin‐deficient mice for phagocytosis assays. As shown in Figure 1B and C, no significant differences were observed between wild‐type and mindin‐deficient macrophages in phagocytosis of chicken red blood cells (CRBCs) or fluorescent particles and the macrophages from mindin‐deficient mice were defective in their ability to phagocytize *E  coli* compared with those from wild‐type mice (Figure S7A). This is consistent with the reports from He et al.^16^ Although we counted at least 200 macrophages per sample under oil‐immersion microscopy, and this method could not efficiently detect the phagocytosis. Therefore, we labelled the bacteria with FITC (Figure S2) and performed FCM to detect peritoneal macrophage phagocytosis. Our results indicate that phagocytosis of bacteria was significantly (Figure S5A) decreased in mindin knockout macrophages (Figure 1E). Furthermore, the addition of recombinant mindin was sufficient to restore the phagocytosis ability of mindin‐deficient macrophages and further enhanced their phagocytic capacity towards *E  coli* compared with the baseline wild‐type macrophages (Figure 1E). In addition, no significant (Figure S5B) differences in phagocytosis of fluorescent particles were observed in either group (Figure 1D). These results agree with previous reports on mindin, which have shown that mindin only clears pathogens such as bacteria or viruses,^15^ but the involved mechanism needs to be further investigated.

### 3.2 Mindin binds with immunogens to promote phagocytosis

**Figure 2:**
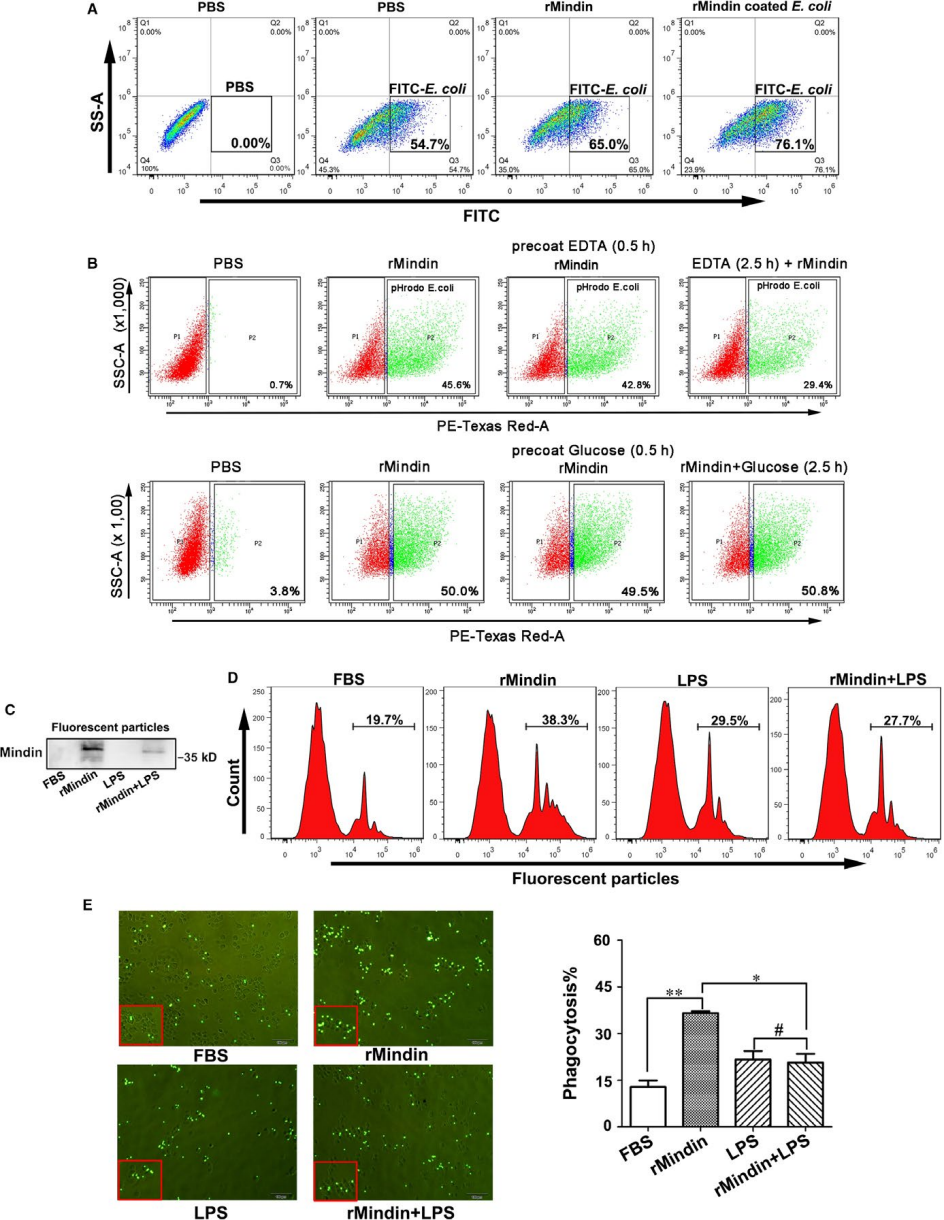
Mindin binds with immunogens to promote phagocytosis. (A) RAW264.7 macrophages were treated with PBS, FITC‐*E  coli* or rMindin (1 µg) and were then analysed with flow cytometry. (B) pHrodo‐*E  coli* and rMindin were pre‐treated with PBS, EDTA (4 mmol/L) or glucose (6 mmol/L), and phagocytosis was analysed using flow cytometry. (C) Fluorescent particles were pre‐treated with FBS, rMindin or LPS, and binding with rMindin was analysed with Western blotting. The RAW264.7 cell phagocytosis of fluorescent particles was analysed with flow cytometry (D) or fluorescence microscopy (E, left panel). The data are presented as the percentage of fluorescent particles that were phagocytosed among all the groups and are shown as the means ± SD from three separate experiments. *P < 0.05, **P < 0.01, #no significant difference (E, right panel)

Mindin has been reported to directly bind to bacteria and their components, such as lipopolysaccharide (LPS) and lipoteichoic acid (LTA).^16^, ^17^ To determine whether mindin enhances macrophage‐mediated elimination of microbial pathogens by binding bacteria, we pre‐incubated recombinant mindin with FITC‐labelled bacteria for 30 minutes, added the mixture to RAW264.7 cells, and performed a phagocytosis assay. As shown in Figure 2A, phagocytosis of *E  coli* that was pre‐coated with recombinant mindin was significantly (Figure S5C) increased compared with the controls, suggesting that mindin may bind *E  coli* first and enhance macrophage phagocytosis. In order to further confirm the authenticity of data, we used the pHrodo‐labelled *E  Coli* to perform phagocytosis experiments. As shown in the Figure S6A, the results are consistent with the effect of FITC‐labelled *E  coli*. It was reported that the bacterial agglutination by mindin is calcium dependent and involve a carbohydrate recognition.^16^ To further examine whether the combination of mindin and *E  coli* is calcium dependent or dependent on carbohydrate recognition, we added EDTA and glucose to the mixture of rMindin and *E  coli*. As shown in the upper panel of Figure 2B, the phagocytosis of mindin was not affected by the pre‐incubation of EDTA instead of co‐incubation. However, there were no significant differences in phagocytosis in glucose group (Figure 2B lower panel), indicating that mindin binding with *E  coli* may be Ca^2+^ or Mg^2+^dependent and not carbohydrate recognition. We envision whether mindin can enhance phagocytosis after binding to other immunogens and we cannot rule out certain components of bacteria that enhance phagocytosis, so we now repeat this experiment using fluorescent particles. We pre‐incubated fluorescent particles with rMindin, FBS or LPS and performed the binding assay. As shown in Figure 2C, rMindin can bind with fluorescent particles, and this binding was blocked by LPS. Thus, we further performed the phagocytosis assay, and as shown in Figure 2D and E, mindin enhanced the RAW264.7 macrophage‐mediated phagocytosis of fluorescent particles, and LPS significantly blocked this phagocytosis. Furthermore, mindin binds with immunogens to promote phagocytosis in a dose‐dependent manner (Figure S3).

### 3.3 Mindin binds with integrin Mac‐1

**Figure 3:**
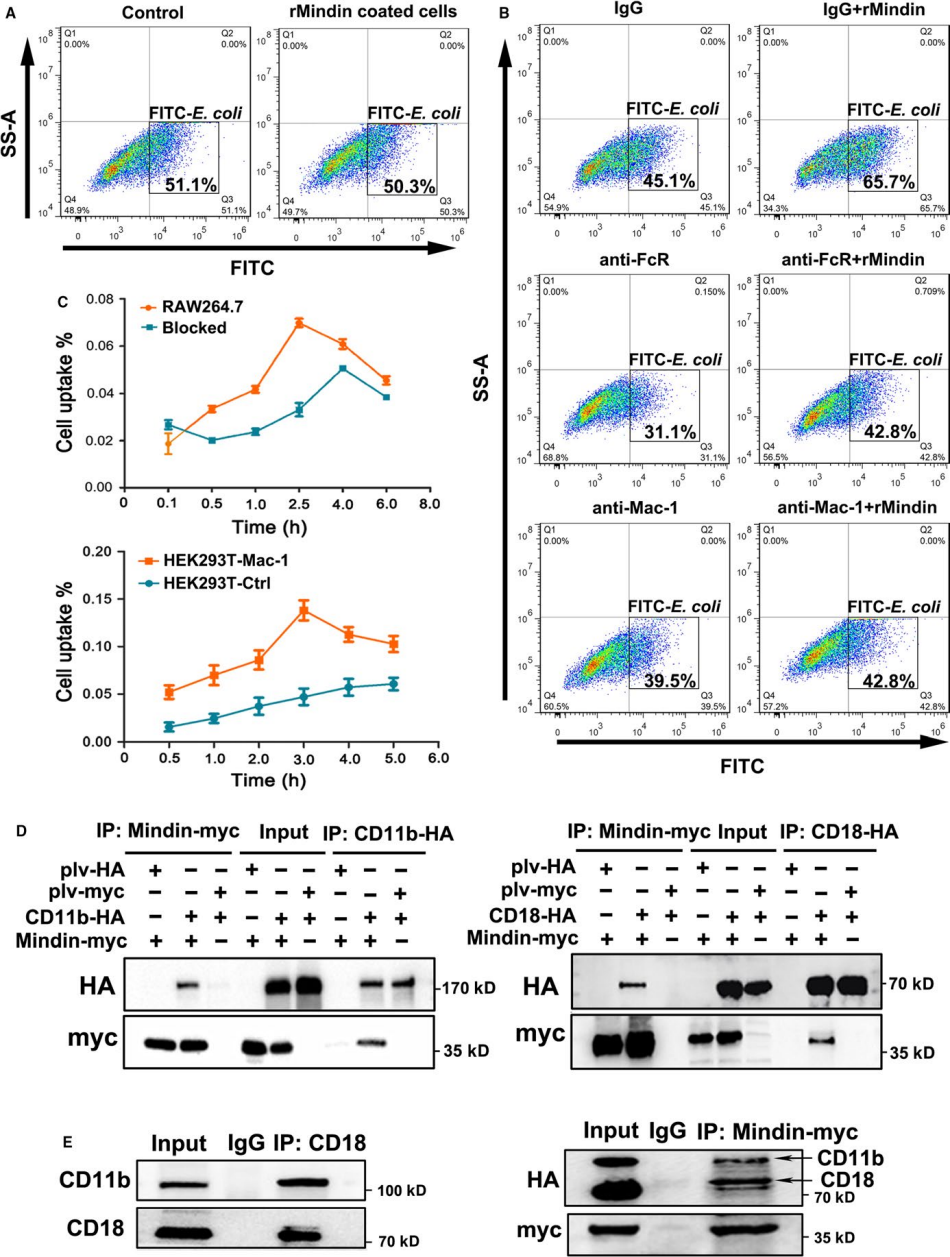
Mindin binds with integrin Mac‐1. (A) RAW264.7 macrophages were pre‐treated with rMindin and were analysed for phagocytosis of *E  coli*. The negative control is presented in Figure S6B (B) FITC‐labelled *E  coli* pre‐treated with rMindin were compared with the control group, and neutralizing antibodies against FcR and Mac‐1 were employed during the phagocytosis assay. The negative control is consistent with Figure 3A. (C) RAW264.7 cells or stably expressing Mac‐1 cells were added to 131I‐mindin and counted with a γ–counter. The percentage of cell uptake was calculated, and the values are expressed as the means ± SD. Blockade indicates the anti‐Mac‐1 group in Raw264.7 cells. (D) Immunoprecipitation assays were performed in HEK293T cells using anti‐HA or anti‐Myc antibodies, and the results were analysed via Western blotting with anti‐HA or anti‐Myc antibodies. (E) Immunoprecipitation assays were performed in HEK293T cells using an anti‐CD18 or anti‐Myc antibody, and the results were analysed by Western blotting with anti‐CD18, anti‐CD11b, anti‐HA or anti‐Myc antibodies

**Figure 4:**
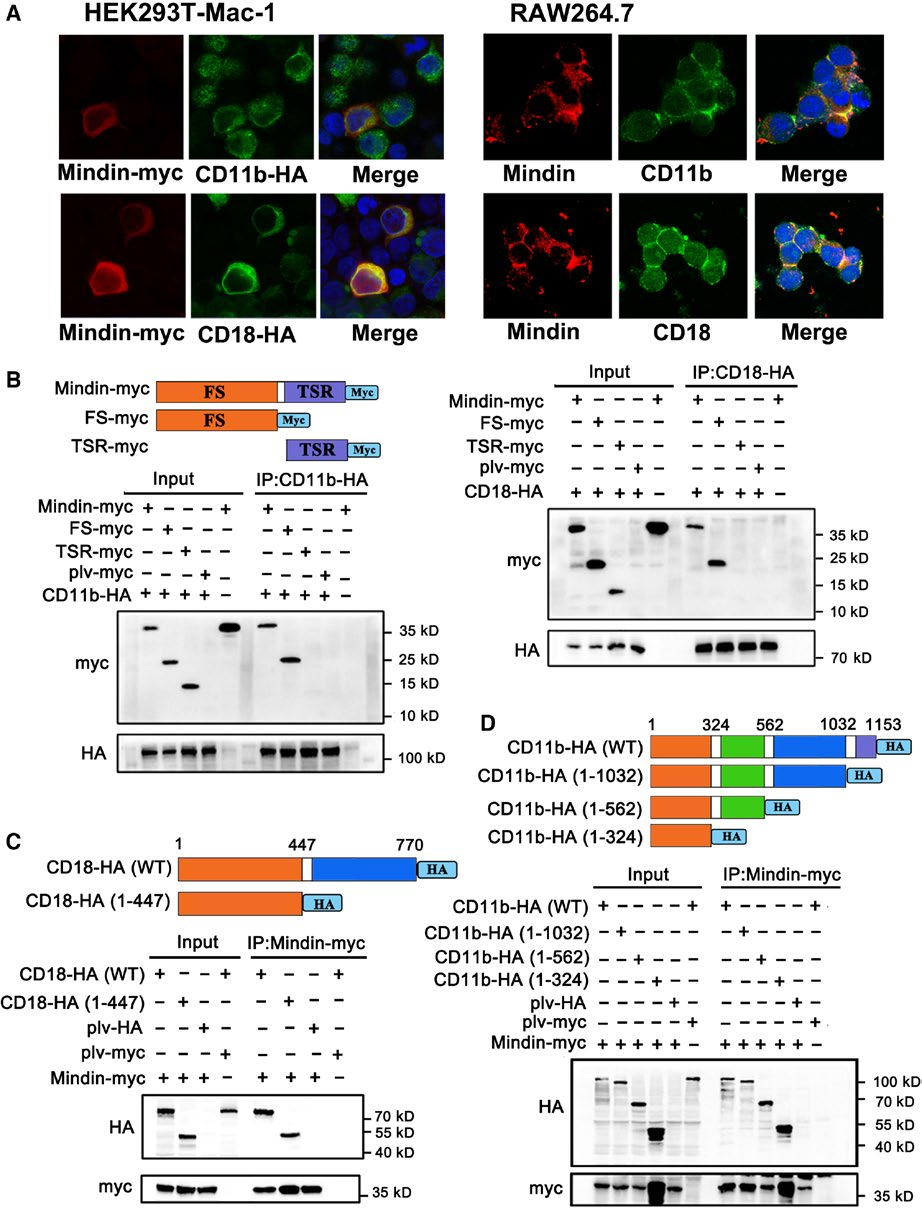
Mindin binds with integrin Mac‐1. (A) HEK293T cells stably expressing Mac‐1 were transfected with mindin‐Myc and stained with anti‐Myc or anti‐HA antibodies (left panels). RAW264.7 macrophages were pre‐treated with mindin for 12 h and then stained with anti‐mindin, anti‐CD11b and anti‐CD18 antibodies (right panels). B‐D, Immunoprecipitation assays were performed using mouse anti‐HA or anti‐Myc antibodies, and the results were analysed by Western blotting with rabbit anti‐HA or anti‐Myc antibodies

To investigate the function of mindin on macrophages, we pre‐treated RAW264.7 macrophages with rMindin, washed the cells, mixed them with bacteria and then performed a phagocytosis assay. As shown in Figure 3A, no significant (Figure S5E) differences were observed between the cells pre‐treated with rMindin and control group cells, which indicates that mindin does not directly activate macrophage phagocytosis. Therefore, mindin may act as an opsonin. To test this hypothesis and to determine the potential opsonic receptor for mindin, we pre‐treated FITC‐labelled *E  coli* with rMindin and employed neutralizing antibodies against FcR and Mac‐1, which are well‐known opsonic receptors, in the phagocytosis assay. Interestingly, neutralizing both FcR and Mac‐1 efficiently blocked phagocytosis compared with that in the control group. However, the addition of rMindin rescued the anti‐FcR‐induced decrease in phagocytosis and failed to rescue the anti‐Mac‐1‐induced phenotype (Figure 3B). These results suggest that mindin might be interacting with Mac‐1 rather than FcR, which is supported by a previous report demonstrating through binding kinetics that mindin acts via integrin binding.^17^ To define the binding strength of mindin with its receptor, radioactive isotope ^131^I‐labelled mindin was prepared (Figure S4A). The cell uptake assay showed that mindin can bind to RAW264.7 macrophages that express endogenous Mac‐1 and can also bind to HEK293T cells stably expressing Mac‐1 (Figure 3C). The cell uptake percentage of ^131^I‐mindin increased over time and peaked at 2.5‐3 h in each experiment, and the anti‐Mac‐1 antibody significantly (Figure 3C) blocked this affinity in RAW264.7 macrophages. To further determine the distribution of ^131^I‐mindin in vivo, we constructed a xenograft model, and free ^131^I‐mindin was more highly concentrated in the Mac‐1‐overexpressing xenograft tissue than in the control tissue (Figure S4B and C). These results indicate that mindin has a strong affinity for integrin Mac‐1 in vitro and in vivo. We constructed overexpression vectors and performed a co‐IP experiment in HEK293T cells, and our data demonstrated that mindin binds CD11b and CD18 receptors in both forward and reverse co‐IP experiments (Figure 3D). Furthermore, ectogenic co‐IP analysis showed that CD11b and CD18 interact as heterodimers and bind to mindin protein (Figure 3E). Confocal immunofluorescence microscopy analysis showed co‐localization of mindin with ectogenic Mac‐1 on the HEK293T cell surface and endogenous Mac‐1 on the RAW264.7 macrophages membrane (Figure 4A). To further determine the domain of mindin that binds to Mac‐1, we constructed plasmids containing either FS‐Myc or TSR‐Myc fragments and full length mindin. Our co‐IP analysis confirmed that the FS domain of mindin binds to CD11b and CD18, while the TSR terminus could not (Figure 4B). Furthermore, to define the domain of CD11b/CD18 that combines with mindin, we designed plasmids encoding fragments of CD11b and CD18. As shown in Figure 4C and D, mindin binds with the 1‐324 amino acid fragment of CD11b and the 1‐447 fragment of CD18. These results agree with previous reports that FS domain interact with αM‐I region of CD11b/CD18,^17^ while whether the interaction involves in bacterial phagocytosis is still unknown.

### 3.4 Blockade of the α_M_‐I domain of Mac‐1 decreases mindin‐induced phagocytosis

**Figure 5:**
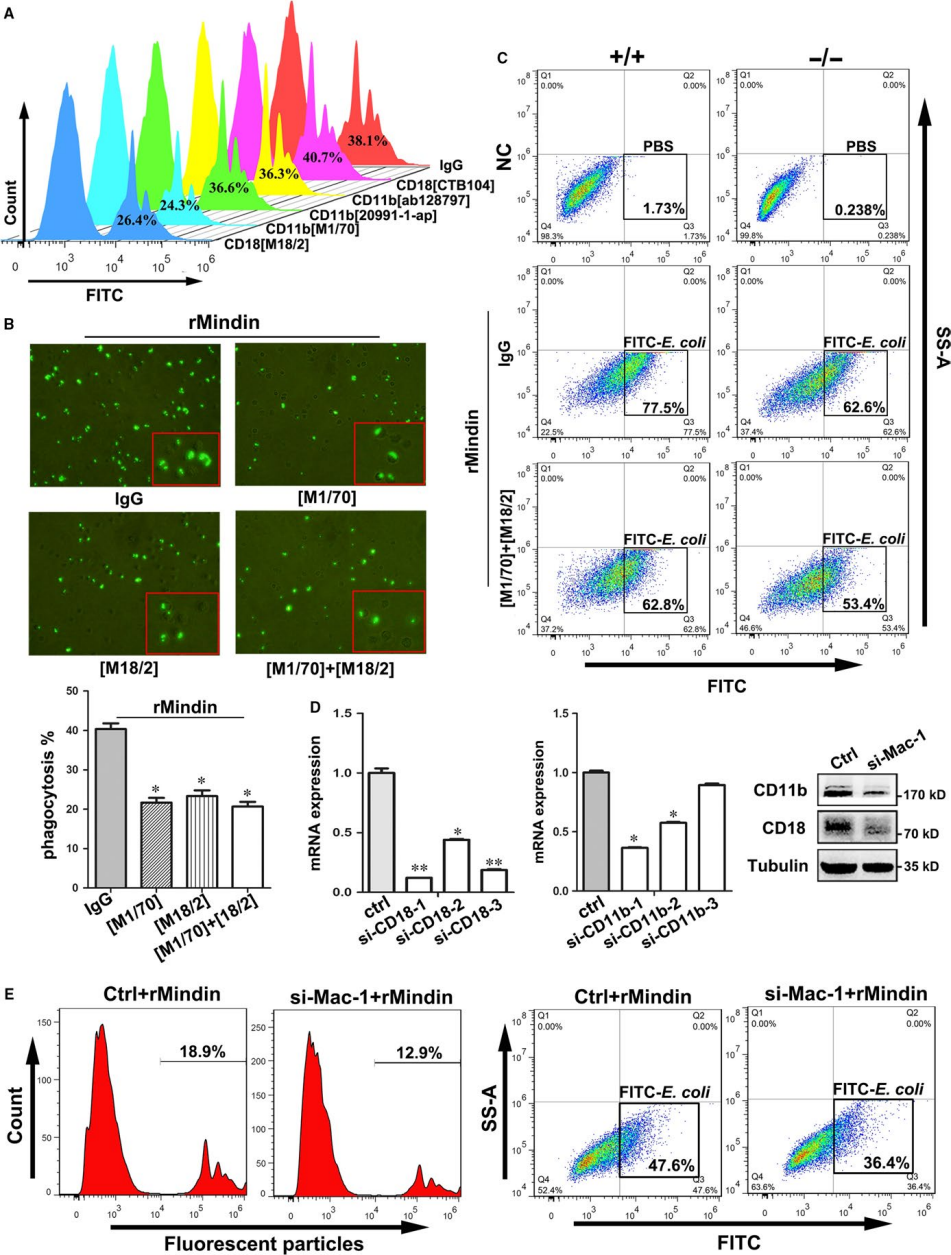
Blockade of Mac‐1 decreases mindin‐induced phagocytosis. (A) RAW264.7 macrophages were pre‐treated with neutralizing antibodies: CD11b ([M1/70], ab128797, 20991‐1‐AP), CD18 ([M18/2], CTB104) and phagocytosis assays were performed using flow cytometry or cells were examined by fluorescence microscopy (B, lower panel for quantitative analysis of upper images). (C) Peritoneal macrophages obtained from wild‐type and Mindin‐/‐ mice were pre‐treated with neutralizing antibodies, and phagocytosis assays were performed and analysed using flow cytometry. (D) The silencing effects of siRNA on Mac‐1 were confirmed by RT‐PCR and Western blot analysis. (E) The phagocytosis assay results for fluorescent particles and *E  coli* in Mac‐1 silenced RAW264.7 cells are shown. The negative control is presented in Figure S6C

To investigate whether mindin‐induced phagocytosis requires integrin Mac‐1 involvement, we employed blocking antibodies targeting different regions of CD11b and CD18. Interestingly, anti‐CD11b and anti‐CD18 antibodies which against the α_M_‐I domain^22^, ^23^ strongly inhibited mindin‐induced phagocytosis of fluorescent particles, while the other antibodies did not show significant blocking effects (Figure 5A and B), suggesting that mindin‐induced phagocytosis requires interaction with the α_M_‐I domain of Mac‐1. We isolated peritoneal macrophages from wild‐type and mindin‐deficient mice and performed the *E  coli* phagocytosis assays. As shown in Figure 5C, mindin‐deficient macrophages showed decreased phagocytosis, and anti‐CD11b and anti‐CD18 antibodies inhibited the *E  coli* phagocytosis that was induced by mindin. Furthermore, we designed siRNA to target the sequences of CD11b and CD18 and transferred the si‐CD18‐1 and si‐CD11b‐1 RNA into the cells and performed a Western blot (Figure 5D). As shown in Figure 5E, phagocytosis of fluorescent particles or *E  coli* was significantly decreased when the expressions of CD18 and CD11b were silenced in RAW264.7 macrophages. Taken together, blockade of the α_M_‐I domain of Mac‐1 decreased mindin‐induced phagocytosis.

### 3.5 Mindin induced Syk family phosphorylation and NF‐κB p65 translocation into the nucleus

**Figure 6:**
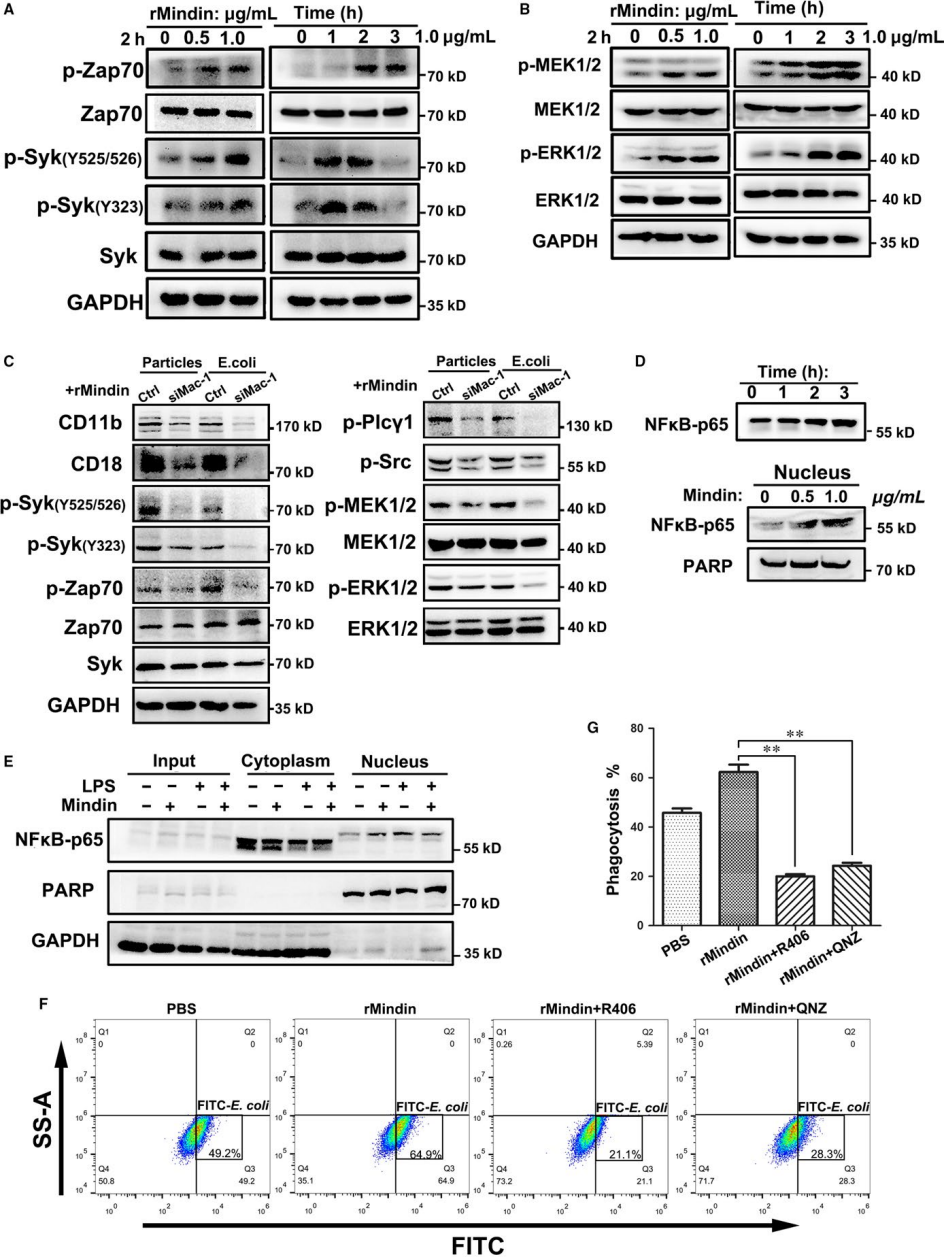
Mindin induces phosphorylation of the Syk family and translocation of NF‐κB p65 into the nucleus. (A) The Western blot analysis results of the phosphorylation levels of Syk family kinases are shown at different time points and concentration gradients. (B) Western blot analysis results are shown for the MAPK pathway at different time points and concentration gradients. (C) We extracted protein from the cells used for the phagocytosis assay shown in Figure 5E and performed Western blot analysis of the Syk and MAPK pathways. (D and E) Western blot analysis results of the p65 levels in nuclear extracts are shown; LPS was employed as a positive control. These experiments were performed three times and selected the representative data. (F) RAW264.7 macrophages were pre‐treated with R406 or QNZ and phagocytosis assays were performed using flow cytometry. The negative control is presented in Figure S6D. (G)The data are presented as the percentage of FITC‐*E  coli* that were phagocytosed among all the groups and are shown as the means ± SD from three separate experiments **P < 0.01.

We aimed to define the signalling pathways involved in mindin and Mac‐1 activities. The spleen tyrosine kinase Syk is known to be phosphorylated and activated upon β2 integrin‐mediated adhesion.^24^, ^25^ To determine whether Syk is involved in mindin‐induced phagocytosis, we treated RAW264.7 cells with rMindin and, as shown in Figure 6A, the phosphorylation levels of Syk and the ζ‐chain associated protein kinase of 70 kDa (Zap‐70) were increased in a concentration‐ and time‐dependent manner. Mindin also activated the MAPK pathway, including MEK1/2 and ERK1/2 (Figure 6B), which are induced by a variety of extracellular stimuli. We extracted protein from the cells in the phagocytosis assay employed in Figure 5E and performed a Western blot analysis. As shown in Figure 6C, when Mac‐1 was silenced by siRNA, the phosphorylation levels of Syk, Zap‐70, MEK1/2, ERK1/2, Src and Plcγ1 were decreased, which suggests that mindin interacts with Mac‐1 and thereby activates the Syk family and the MAPK pathway. Our previous study showed that mindin may induce NF‐κB promoter activation in a TLR‐9‐mediated manner during acute intestinal inflammation.^26^ Herein, we investigated whether mindin could activate NF‐κB in macrophages. We observed that NF‐κB p65 was up‐regulated in a time‐dependent manner in RAW264.7 macrophages treated with mindin (Figure 6D, upper panel). Furthermore, nuclear extraction experiments demonstrated that mindin directly induced NF‐κB p65 translocation into the nucleus (Figure 6D lower panel and E). To define the activation of the Syk pathway and NF‐κB translocation are required for the enhanced phagocytic activity, we inhibit the phosphorylation of Syk and NF‐κB p65 by inhibitors. As shown in Figure 6F and G, phagocytosis of the groups that add Syk inhibitor R406 and NF‐κB inhibitor QNZ were significantly decreased compared with the others, suggesting that mindin may activate the Syk pathway and NF‐κB translocation to enhance macrophage phagocytosis.

## 4 DISCUSSION

Macrophages are able to remove unwanted particulate matter, including a large variety of opportunistic and pathogenic bacteria, fungi and protists; polluting debris, such as asbestos and silicate particles and endogenous cellular components, such as apoptotic and senescent cells.^4^ In our study, mindin‐deficient macrophages did not show defective phagocytosis towards CRBCs or particles. However, rMindin binding to the particles promoted phagocytosis of the particles. These data indicate that mindin induces phagocytosis by binding directly to immunogens, including particles and bacteria. It has reported that mindin recognizes LPS through its TSR domain,^17^ which provides evidence that the TSR of mindin binds to the LPS of bacteria. Previous studies have shown that agglutination of *Salmonella  typhimurium* with mindin was inhibited by EDTA and glucose and have shown that agglutination by mindin depends on calcium and carbohydrate recognition.^16^ In this study, the phagocytosis of mindin was also inhibited by EDTA but not glucose. The involvement of Ca^2+^ or Mg^2+^ is required during the phagocytosis of mindin. If have chelated Ca^2+^ or Mg^2+^ before, it will not affect the phagocytosis of mindin. Suggesting that Ca^2+^ or Mg^2+^ only play a role in the process of phagocytosis and receptors that bind to mindin need the participation of Ca^2+^ or Mg^2+^ and recognition of mindin and bacteria is not a simple carbohydrate way.

Mindin has the ability to recruit macrophages to inflammation sites.^18^ We envisage the mindin‐mediated macrophage phagocytosis process as follows: macrophages are recruited to a site of abundant mindin, which combines with immunogens, and phagocytize the complex by binding to the opsonic receptor. Receptors for the Fc portion of immunoglobulin G (IgG) and integrin Mac‐1 are particularly effective at recognizing extraneous antigens.^27^, ^28^ They function as opsonic receptors. We determined that mindin interacts with integrin Mac‐1 rather than FcR and thereby promotes macrophage phagocytosis. The mindin‐induced phagocytosis of bacteria was attenuated when we applied neutralizing antibodies targeting Mac‐1 or silenced the expression of CD11b/CD18 via siRNA. Here we also confirm that the FS domain of mindin directly binds to the α_M_‐I domain of Mac‐1 by co‐IP, others based on binding kinetics.^17^, ^18^ The integrin ‘head' consists of the seven‐blade propeller of the α‐subunit that makes intimate contact with the GTPase‐like domain of the β‐subunit.^29^ The α_M_‐I domain, which is inserted between two loops on the upper surface of the propeller, plays a central role in ligand binding.^30^, ^31^, ^32^ The I domains of the β2 integrins contain the Mn^2+^ and Mg^2+^ cation binding MIDAS motif.^33^, ^34^ However, the FS domain of mindin contains 22 glutamate or aspartate residues on its surface that could potentially ligate metals at the MIDAS.^17^ Unlike integrins without I domain (αVβ3, αIIbβ3), β2 integrins contains various sequences, such as GPR, P1 peptide and P2 peptide sequences, as the direct binding subunit.^10^ I domains are highly homologous and highly selective for particular sets of ligands. At the same time, it can recognize multiple and structurally unrelated ligands^10^ and now we confirm that mindin can directly bind to the MIDAS motif of pseudo‐I structure of β2. Integrin Mac‐1 has more than 40 reported protein ligands,^10^, ^11^ and herein, we have defined mindin as a novel Mac‐1 ligand.

Syk and the related ZAP‐70 tyrosine kinase are critical components of the signal transduction machinery of Mac‐1 and play an important role in intracellular signal transduction in immune cells.^35^, ^36^ Our results show that mindin was able to activate the Syk family and increase their phosphorylation levels at Tyr323 and Tyr525/526. Phosphorylation at Tyr323, which is located in the SH2‐kinase linker region of Syk, provides a direct binding site for the TKB domain of Cbl.^37^ Phosphorylation at Tyr525/526, which is located in the activation loop of the Syk kinase domain, is essential for Syk function.^38^ Mindin binding to Mac‐1 increased the Src‐family‐mediated phosphorylation of ITAM‐bearing receptor sidechains and recruited Syk or ZAP‐70 to the receptor complex. The latter kinases are responsible for further downstream signalling through pathways such as the phospholipase C‐g1 and MAPK pathways (MEK1/2 and ERK1/2). The Syk signal path was required in the β2 integrin‐induced signalling leading to the potentiation of the oxidative burst.^25^, ^35^ Midnin may potentiated the oxidative burst of macrophages and facilitated the local reorganization of the cytoskeleton which is of predominant importance for the efficient elimination of invaded bacteria. Moreover, our results demonstrated that the activation of NF‐κB in mindin‐stimulated macrophages was significantly increased, and more p65 was translocated into the nucleus. As a result, p65 bind to DNA and induce inflammatory factor‐related gene transcription and cytokine production like TNF and IL‐6.

In summary, when the body is in the acute phase of bacterial invasion, a large number of mindin are released. Mindin recognizes microbial pathogens and binds to Mac‐1 to enhance phagocytosis in macrophages, eliminate invasion microbial pathogens. Also in the state in which the body is in an excessive state of inflammation, inhibition of the expression of mindin can suppress shock caused by excessive reaction of the body.

## CONFLICT OF INTEREST

The authors declared no conflicts of interest.

## AUTHORS' CONTRIBUTIONS

Guleng B and Ren JL designed the experiments; Liu YS, Wang LF, Cheng XS, Huo YN, Ouyang XM, Liang LY, Lin Y and Wu JF performed the experiments; Guleng B and Liu YS wrote the paper.
