# Supplementary figures and images for: The pattern‐recognition molecule mindin binds integrin Mac‐1 to promote macrophage phagocytosis via Syk activation and NF‐κB p65 translocation
Source: J Cell Mol Med. 2019 Mar 14;23(5):3402–16. doi: 10.1111/jcmm.14236 (PMC6484411; doi:10.1111/jcmm.14236)

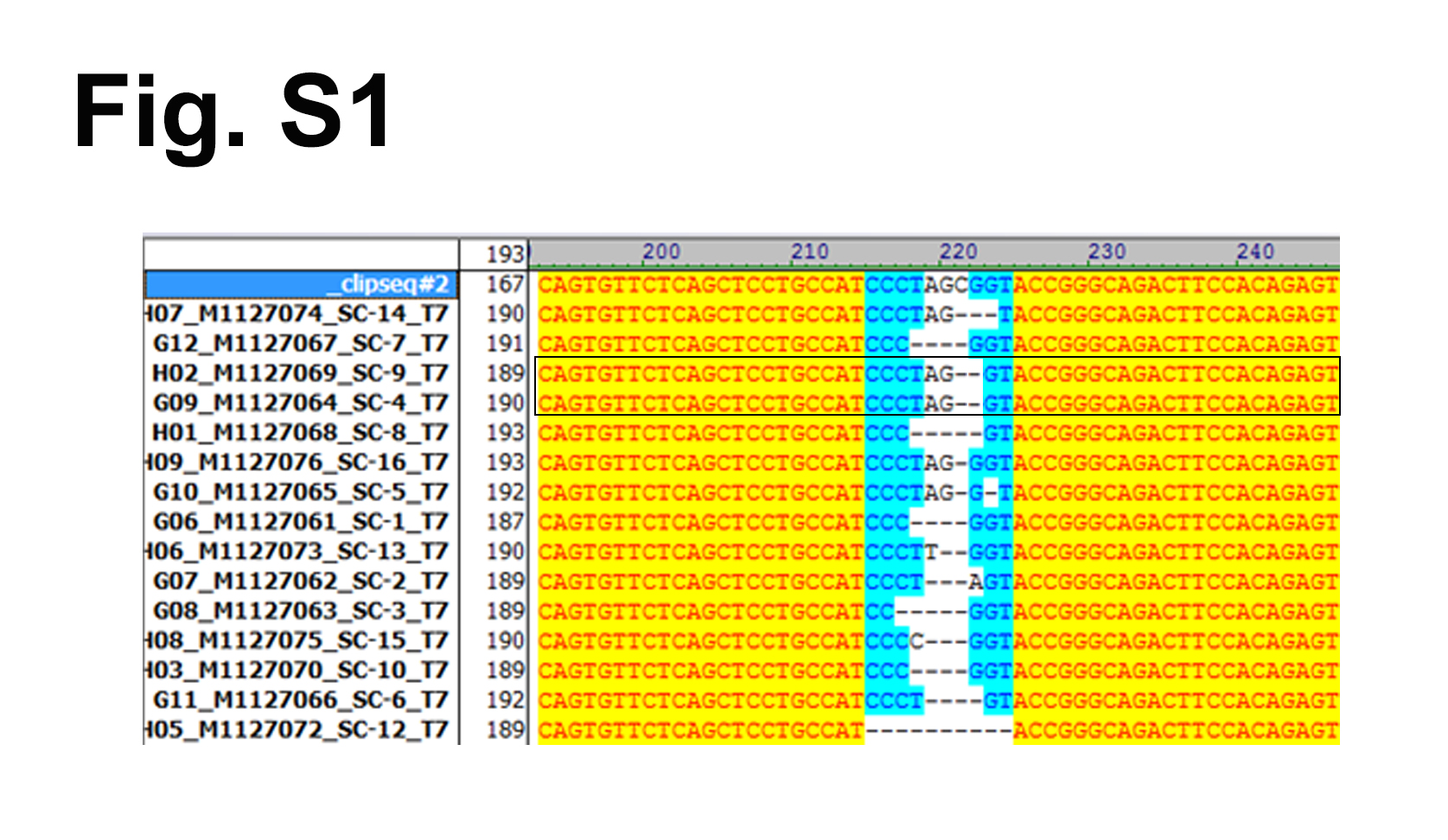

Supplement: Supplementary file 1 [file JCMM-23-3402-s001.jpg]

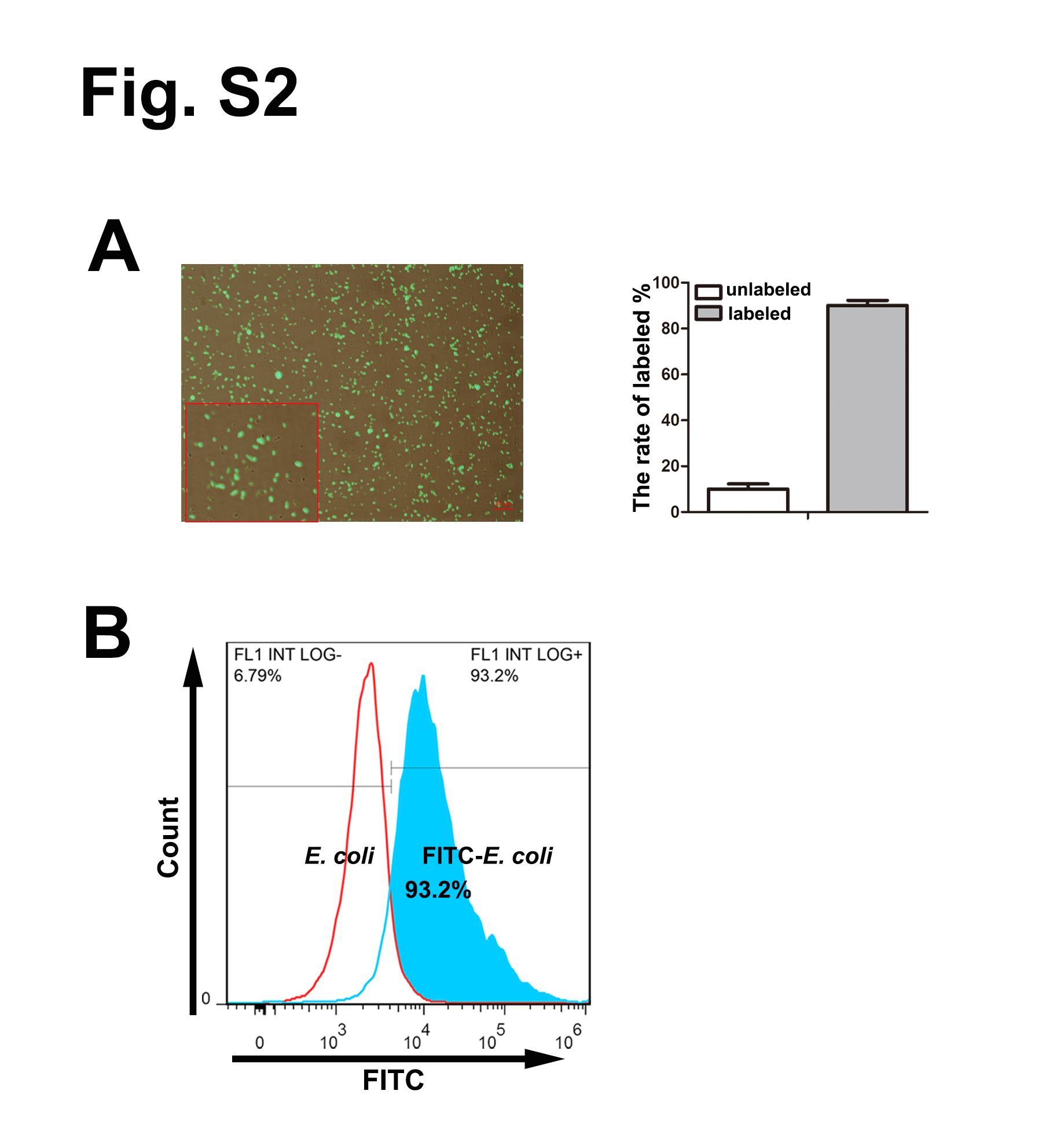

Supplement: Supplementary file 2 [file JCMM-23-3402-s002.jpg]

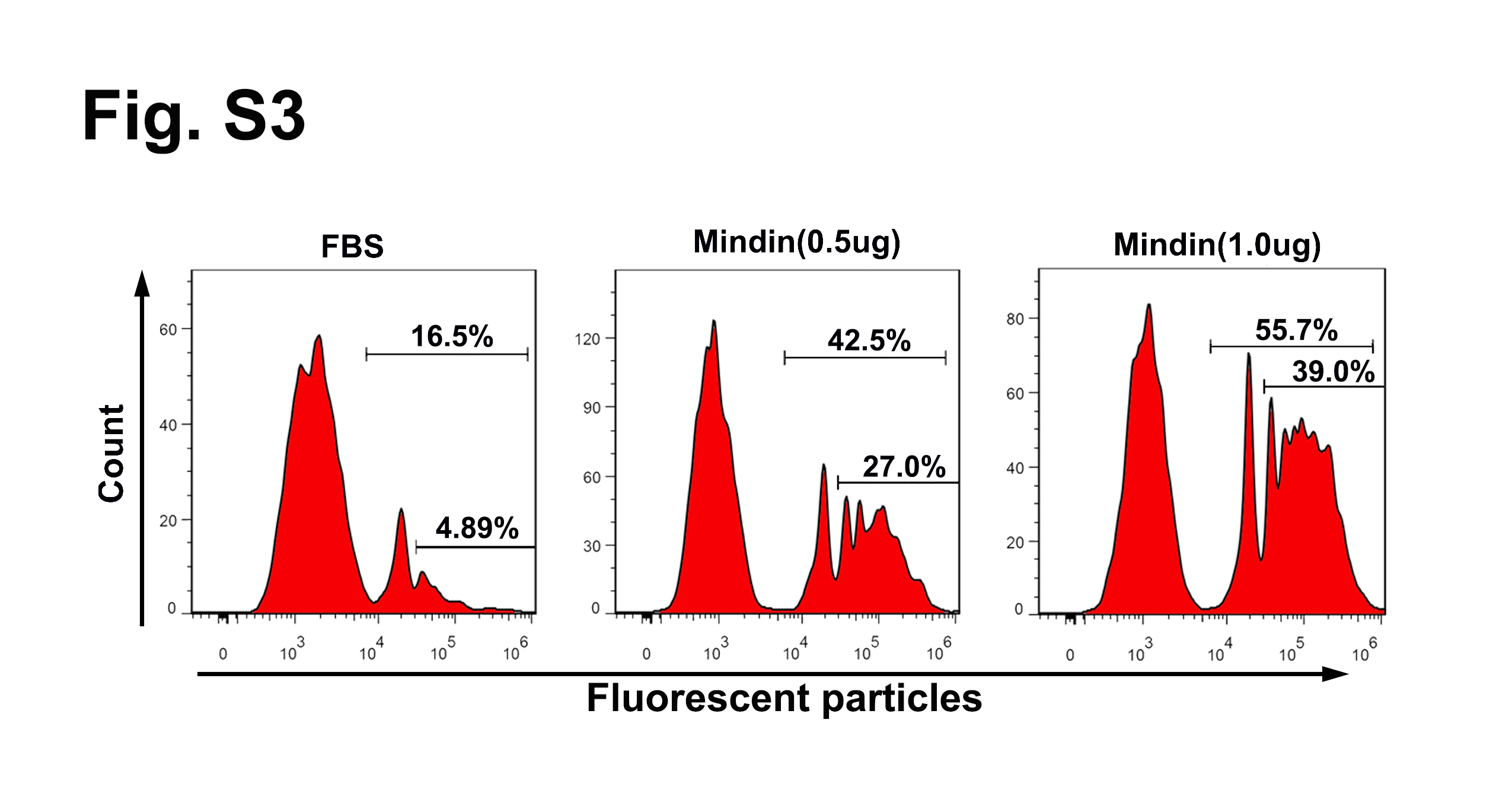

Supplement: Supplementary file 3 [file JCMM-23-3402-s003.jpg]

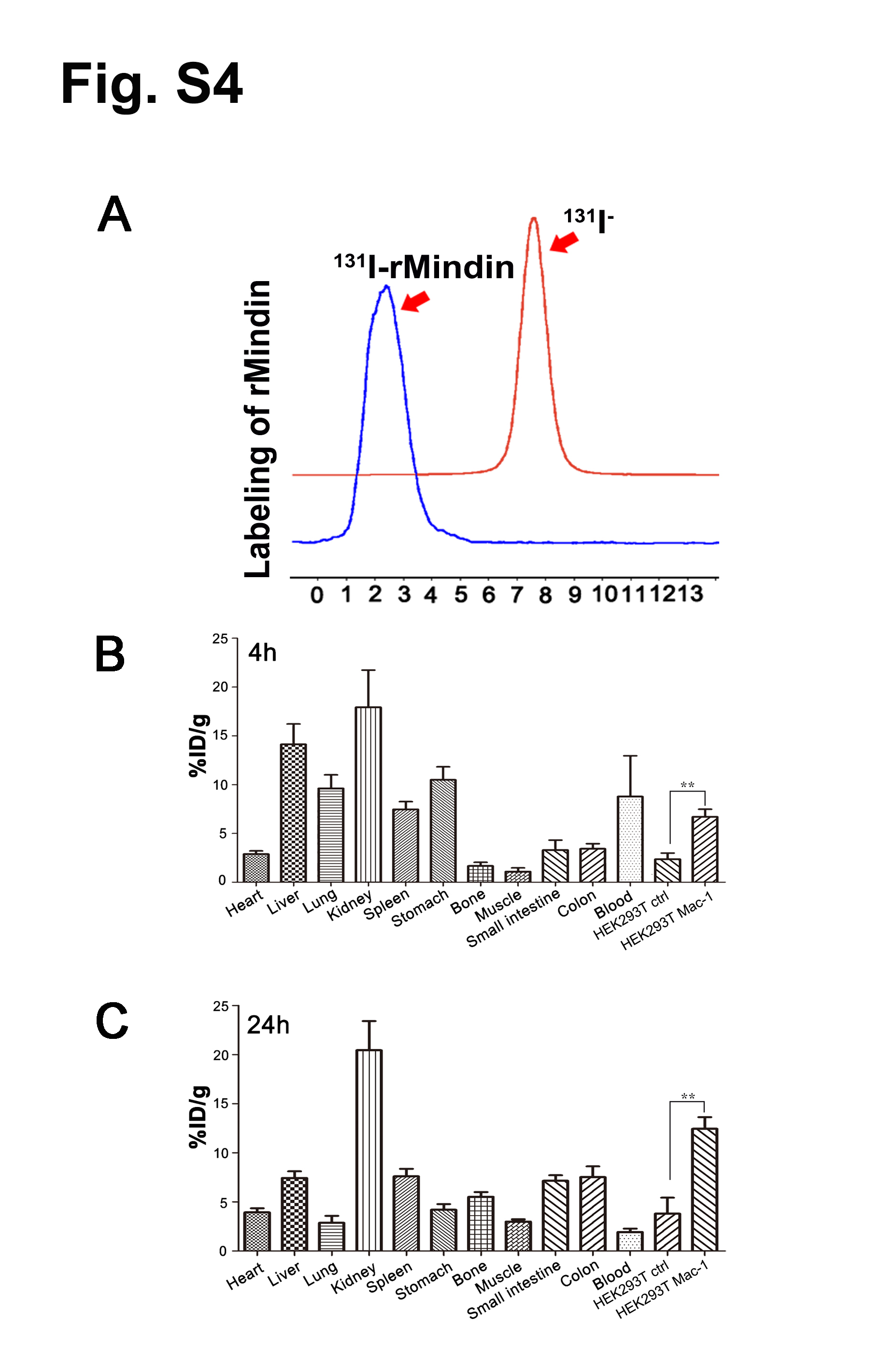

Supplement: Supplementary file 4 [file JCMM-23-3402-s004.jpg]

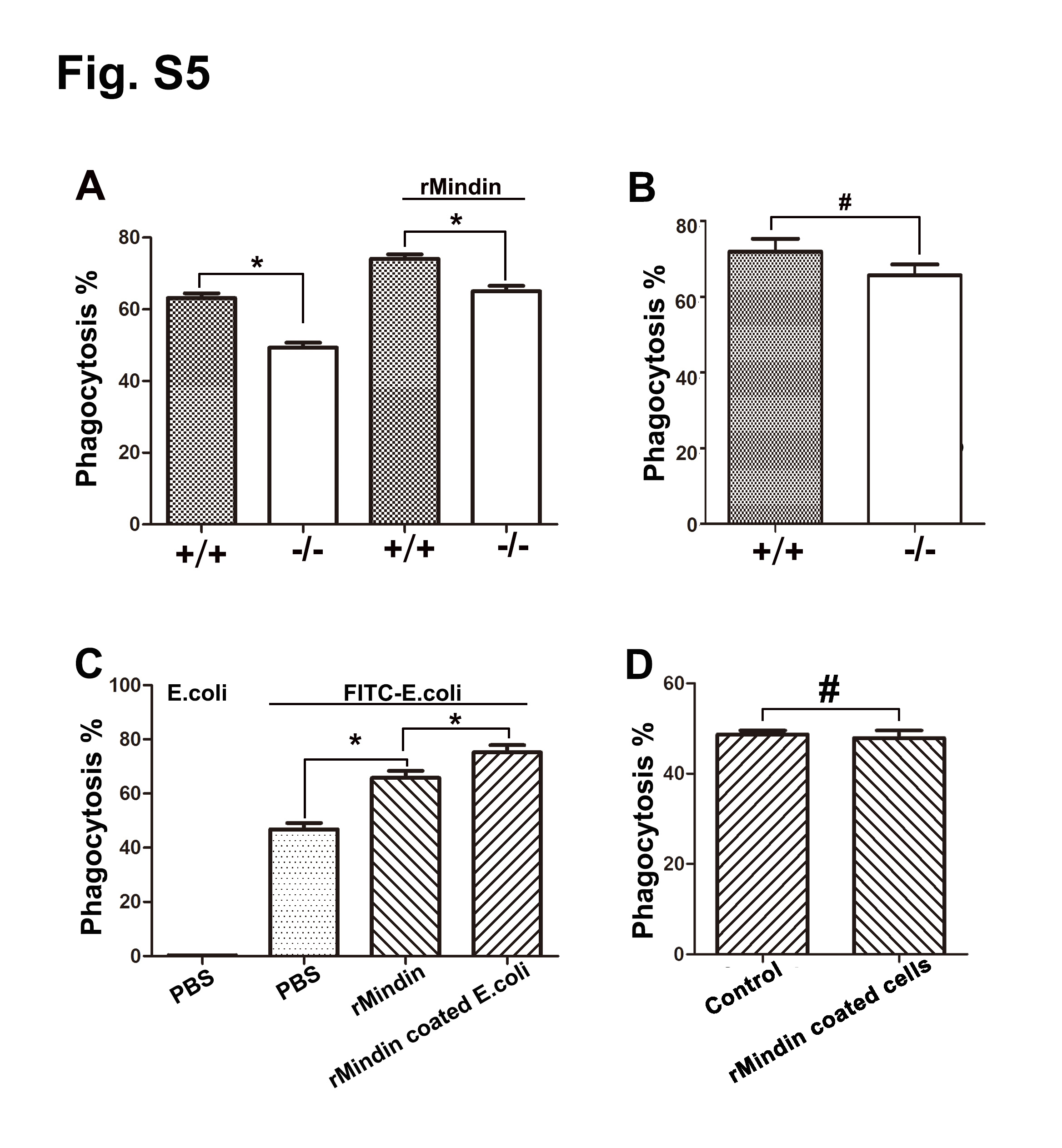

Supplement: Supplementary file 5 [file JCMM-23-3402-s005.jpg]

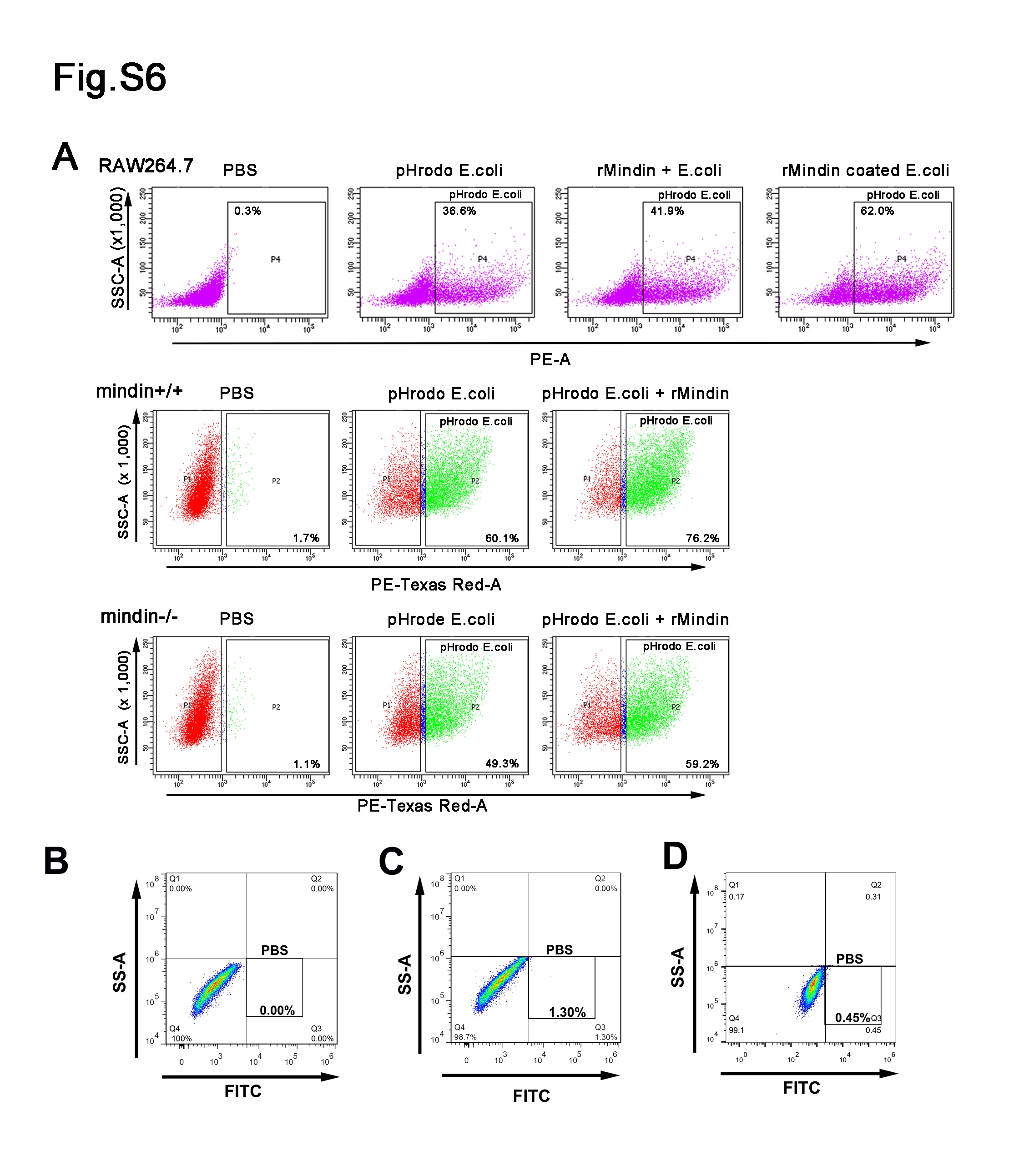

Supplement: Supplementary file 6 [file JCMM-23-3402-s006.jpg]

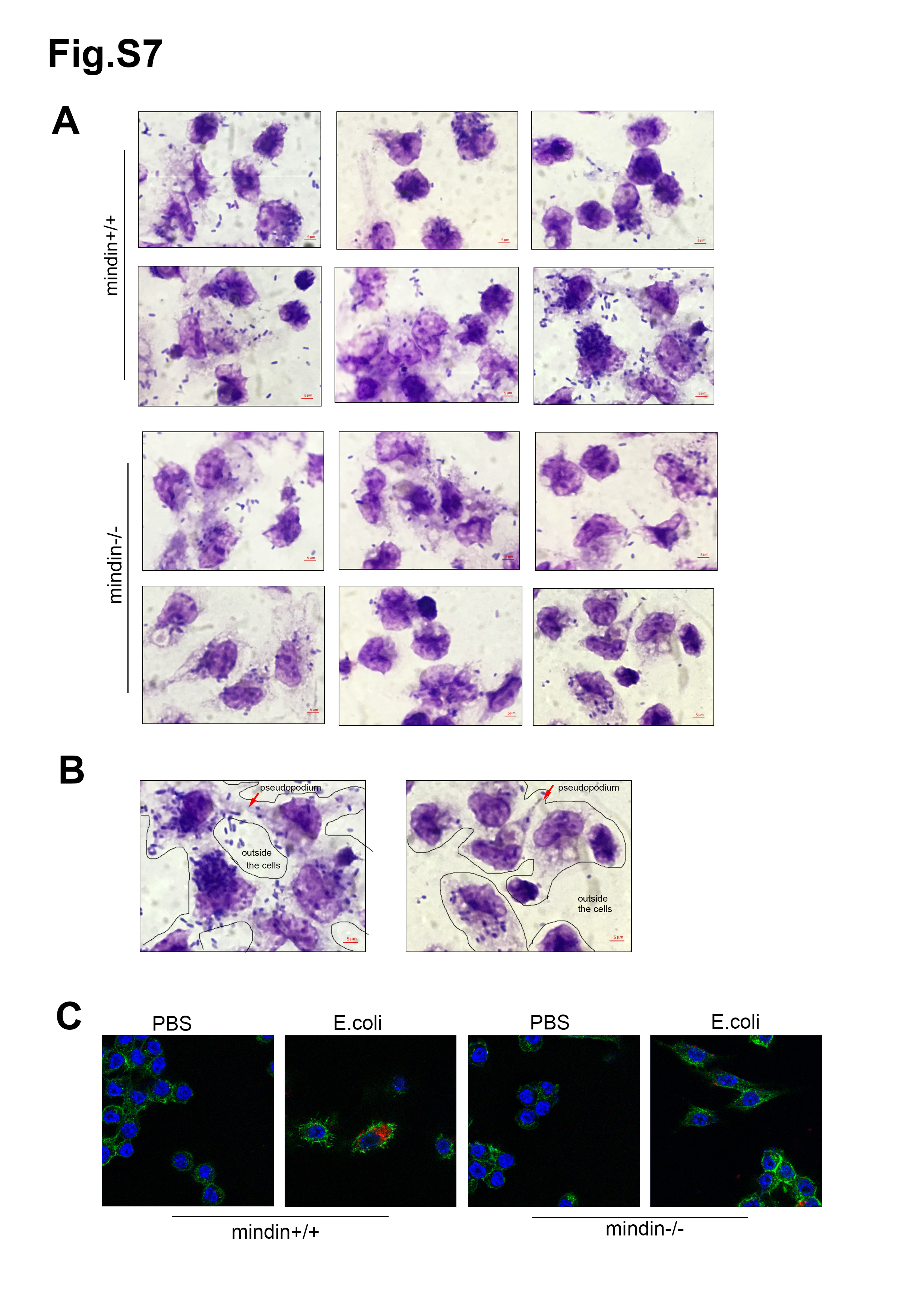

Supplement: Supplementary file 7 [file JCMM-23-3402-s007.jpg]
